# Dietary acid load and chronic kidney disease in elderly adults: Protein and potassium intake

**DOI:** 10.1371/journal.pone.0185069

**Published:** 2017-09-27

**Authors:** Byung-Joon Ko, Yoosoo Chang, Seungho Ryu, Eun Mi Kim, Mi Yeon Lee, Young Youl Hyun, Kyu-Beck Lee

**Affiliations:** 1 Department of Occupational and Environmental Medicine, Kangbuk Samsung Hospital, Sungkyunkwan University School of Medicine, Seoul, Korea; 2 Center for Cohort Studies, Total Healthcare Center, Kangbuk Samsung Hospital, Sungkyunkwan University School of Medicine, Seoul, Korea; 3 Department of Dietetics, Kangbuk Samsung Hospital, Sungkyunkwan University School of Medicine, Seoul, Korea; 4 Department of Biostatistics, Kangbuk Samsung Hospital, Sungkyunkwan University School of Medicine, Seoul, Korea; 5 Division of Nephrology, Department of Internal Medicine, Kangbuk Samsung Hospital, Sungkyunkwan University School of Medicine, Seoul, Korea; Nagoya University, JAPAN

## Abstract

**Background:**

Dietary net endogenous acid production (NEAP), which represents total dietary load of nonvolatile acid, may affect kidney function. Estimated NEAP (eNEAP) is calculated indirectly by the ratio of protein and potassium intake. A few studies are available assessing the association between eNEAP and chronic kidney disease (CKD), and its relation to dietary protein and potassium intake in the elderly.

**Methods:**

A total 1,369 community-dwelling elderly Koreans in the Kangbuk Samsung Cohort Study (KSCS) were evaluated using a food frequency questionnaire (FFQ) and comprehensive health examination. We evaluated the association between eNEAP and the CKD. We also examined their relation to protein and potassium intake.

**Results:**

eNEAP was correlated with potassium intake (r = -0.410, *P* < 0.001), but was not correlated with protein intake (r = -0.004, *P* = 0.879). In a full multivariate adjustment for sociodemographic factors, dietary factors, and comorbidities, the participants with higher eNEAP quartiles (Q2, Q3, Q4) had higher odds of CKD compared to the lowest eNEAP quartile (Q1); OR (95% CI) were 1.47 (0.78–2.72), 1.66 (0.85–3.23), and 2.30 (1.16–4.60) respectively (*P* for trend = 0.019). The odds of CKD decreased for participants with higher potassium intake quartiles (Q2, Q3, Q4) compared to the lowest potassium intake quartile (Q1); OR (95% CI) were 0.52 (0.28–0.95), 0.50 (0.26–0.96), and 0.50 (0.21–0.99) respectively (*P* for trend = 0.050). Protein intake was not associated with CKD. The association between eNEAP and CKD was similar in subgroup analysis.

**Conclusion:**

Dietary acid load was associated with CKD. Among the nutrients related to dietary acid load, potassium intake was negatively associated with CKD, but protein intake was not associated with CKD in elderly adults.

## Introduction

Chronic kidney disease (CKD) is a public health problem that is characterized by high cost and mortality. CKD is a major determinant of poor health outcomes in patients with diabetes, hypertension, and cardiovascular disease (CVD) [1]. CKD in elderly adults is common and has a major impact on disabilities and death [2]. The optimal diet to prevent development of CKD has not been identified. Recent Kidney Disease Improving Global Outcomes (KDIGO) guideline [3] recommended that adults with CKD restrict individual nutrients, such as protein and sodium, to delay the progression of CKD and prevent clinical complications. Individuals do not consume nutrients or foods in isolation [4] and there need to be more dietary studies about various food patterns to prevent CKD.

Diet can essentially affect acid-base status. The contemporary Western-style diet is potentially acid-producing and may damage the kidney via tubular toxicity of ammonium and activation of the renin-angiotensin system [5]. Net endogenous acid production (NEAP) is determined by acid production by sulfur-containing amino acids from proteins counterbalanced by bicarbonate generation from alkali-containing potassium salts. Estimated NEAP (eNEAP) represents the total dietary load of nonvolatile acid added to the body and can be estimated indirectly by the ratio of protein and potassium intake [6].

Recent studies suggest that metabolic acidosis can contribute to the progression of CKD [7] and high dietary acid load can decline kidney function [8]. Low protein diets are recommended for slowing renal progression in adults with CKD notwithstanding inconclusive results of the Modification of Diet in Renal Disease (MDRD) study [3,9]. Potassium intake is associated with blood pressure reduction and appears to improve vascular function[10], which in turn decreases the risk of CVD [11,12]. MDRD study reported that higher urine potassium excretion as a surrogate for dietary potassium intake was associated with low risk for mortality, but not kidney failure [13]. The association between potassium intake and kidney disease is uncertain. Dietary patterns and habits also vary according to race, region, and age group. It is unclear what the relationship is between the dietary acid load and CKD, and what their relationship is protein and potassium intake in elderly adults. We therefore evaluated the association between eNEAP and CKD in a large sample of community dwelling elderly Koreans. We also examined their relation to protein and potassium intake.

## Subjects and methods

### Subjects

The Kangbuk Samsung Cohort Study (KSCS) is a cohort of Korean adults who underwent a comprehensive health screening examination at the Kangbuk Samsung Total Healthcare Centers in Korea. The primary aim of KSCS is to investigate the causes for the development of current chronic diseases, i.e. cardiovascular diseases, cancer, diabetes, and neuropsychiatric disease. A total 8208 elderly participants (aged 65 years or over) of the KSCS 2011–2014 were included and underwent a comprehensive health examination at a baseline visit from March 2011 to December 2014. This study was approved by the Institutional Review Board of Kangbuk Samsung Hospital. The informed consent requirement for this study was waived by the Institutional Board because researchers only retrospectively accessed a de-identified database for analysis purposes. We excluded 3323 participants who did not complete a food frequency questionnaire (FFQ) and 4872 participants who were not assessed for urine albumin. We also excluded 13 participants due to missing important laboratory data. The final sample size for the analysis was 1,369 participants, all of whom were community-dwelling elderly individuals.

### Dietary assessment

Diet was assessed at the beginning of the health examination using a 103-item self-administered FFQ validated for use in Korea and was designed to capture dietary habits during the previous year [14]. Participants were questioned how often, on average, they consumed each type of food or beverage during the past year. The FFQ had three predefined categories of portion size ranges; small, medium, and large. FFQ had nine predefined categories of frequency, ranging from never or seldom to ≥ 3 times per day for foods, and from never or seldom to ≥ 5 times per day for beverages. Participants were also asked to report the consumption period (i.e. 3, 6, 9, or 12 months) for seasonal consumption of fruits. Total consumption of each of the foods and beverages was calculated by multiplying the frequency of consumption by specific portion sizes. Dietary protein intake in this study was a total of animal sourced protein and plant sourced protein. Total energy and nutrient intake was calculated using the food composition table developed by the Korean Nutrition Society [15].

eNEAP is estimated indirectly using the ratio of protein and potassium intake in the diet, as established by Frassetto et al. [16] eNEAP was estimated from dietary intakes using a previously validated equation: eNEAP (mEq/d) = 54.5 (protein intake [g/d] ÷ potassium intake [mEq/d]) -10.2.

### CKD and covariates

Blood specimens were sampled from the antecubital vein after at least a 10-hour fast. Serum and urine creatinine levels were measured by traceable to an isotope-dilution mass spectrometry reference method using automated chemistry analyzer (Cobas 8000 c702; Roche Diagnostics, Tokyo, Japan). The Chronic Kidney Disease Epidemiology Collaboration (CKD-EPI) equation was used to calculate estimated glomerular filtration rate (eGFR) [17]. Urine albumin was measured by immunoturbidimetric assay using an automated chemistry analyzer (Modular P800; Roche Diagnostics, Tokyo, Japan). Urine albumin-to-creatinine ratio (ACR) was calculated as the ratio between urinary albumin and urinary creatinine and expressed in mg/g. CKD was defined as eGFR < 60ml/min/per 1.73 m^2^ or ACR ≥ 30 mg/g [3].

Data on medical history, smoking habits and educational status were collected using a standardized self-administered questionnaire. Anthropometry data, blood pressure, and blood samples were obtained by trained staff during the health examination. Low education defined that highest level of education is high school or less. Health-enhancing physical activity (HEPA) was defined as more than 150 min/week moderate activity, or 75 min/week vigorous activity, or an equivalent combination according to global recommendations on physical activity for health of World Health Organization [18]. CVD is defined medical history of myocardial infarction, heart failure, peripheral artery disease, or stroke.

### Statistical analysis

Data were expressed as frequency (%) for categorical variables and mean ± standard deviation (SD) for continuous variables. ACR and C-reactive protein were expressed as median and interquartile range, because their distributions were skewed. Clinical and dietary characteristics according to eNEAP quartiles were compared with a Chi-square analysis for categorical variables, the analysis of variance (ANOVA) for continuous variables and a Kruskal-Wallis test for ACR and C-reactive protein.

We constructed multivariate logistic regression models to determine whether eNEAP and its nutrient components of protein and potassium intake were associated with CKD. The dietary intakes of eNEAP, potassium, protein were divided into quartiles. Logistic regression was used to calculated the odds ratio (OR) and its 95% confidence interval (CI) for CKD using the lowest quartile (Q1) of dietary intake as the reference group. Crude and multivariate analyses were adjusted for various CKD risk factors, energy intake and sodium intake. Model 1 was adjusted for age, sex, total caloric intake, and sodium intake. Model 2 was adjusted for the variables in model 1 plus body mass index (BMI), smoking, education status, HEPA, hyperlipidemia, hypertension, diabetes, and CVD. Tests for trend were performed using mean values of intake in the quartile categories as continuous variables in the logistic regression models. A *P* value < 0.05 was considered statistically significant. All statistical analyses were performed using Stata Version 14 (StataCorp LP, College Station, TX, USA).

## Results

### Baseline characteristics

The average age of study participants was 69.0 ± 3.7 years (mean ± standard deviation), and 55.2% of the participants were male. The prevalence of hypertension, diabetes, hyperlipidemia, and CVD was 45.7%, 19.9%, 25.4%, and 11.0%, respectively, and was not different according to eNEAP quartile. There were no differences in BMI, CRP, total cholesterol, triglycerides, or fasting glucose according to eNEAP quartile. The prevalence of CKD, low eGFR (<60 mL/min/1.73 m^2^), and albuminuria (ACR ≥ 30 mg/g) was 15.9%, 7.0%, and 11.1%, respectively. Participants with high eNEAP had more CKD (*P* = 0.044) and low eGFR (*P* = 0.028) (Table 1).

**Table 1:** Clinical characteristics of 1,369 elderly adults by dietary eNEAP quartiles.

Variable	All	eNEAP, mEq/day				*P*
		Q1 (1.1 to 33.3)	Q2 (33.4 to 45.5)	Q3 (45.6 to 60.5)	Q4 (60.6 to 219.1)	
**Number of patients**	1,369	343	342	342	342	
**Age, y**	69.0±3.7	68.9±4.1	68.8±3.2	68.8±3.5	69.2±3.9	0.440
**Male sex, %**	55.2	44.0	53.5	57.6	65.5	<0.001
**Hypertension, %**	45.7	44.6	49.7	40.6	48.0	0.086
**Diabetes, %**	19.9	19.8	20.2	17.0	22.5	0.342
**Hyperlipidemia, %**	25.4	27.7	24.3	24.6	25.2	0.724
**CVD, %**	11.0	10.5	13.9	9.8	11.2	0.769
**Current smoker, %**	10.3	7.7	10.9	8.2	14.4	0.051
**Low educated, %**	63.2	62.8	65.5	64.2	60.1	0.573
**HEPA, %**	29.1	27.9	32.7	27.3	28.8	0.413
**BMI, kg/m**^**2**^	23.8±2.9	23.6±2.7	23.8±2.9	23.8±3.0	23.9±3.1	0.591
**Systolic BP, mmHg**	119±14	120±15	119±14	119±15	119±14	0.826
**Diastolic BP, mmHg**	72±9	72±9	72±9	72±9	72±9	0.809
**CRP, mg/L**	0.6 [0.2 1.0]	0.5 [0.2 1.0]	0.6 [0.2 1.0]	0.6 [0.2 1.1]	0.6 [0.2 1.1]	0.739
**Total cholesterol, mg/dL**	195±38	196±39	196±38	198±37	191±39	0.118
**Triglyceride, mg/dL**	114±58	111±52	116±57	111±57	116±64	0.495
**Fasting glucose, mg/dL**	103±19	104±19	104±21	102±20	103±17	0.578
**eGFR, mL/min/1.73m**^**2**^	79.4±13.0	79.3±13.5	80.5±12.0	80.0±12.2	77.7±13.9	0.028
**eGFR < 60 mL/min/1.73m**^**2**^**, %**	7.0	6.1	5.8	6.4	9.7	0.175
**Urinary ACR, mg/g**	7.6 [4.4 15.0]	7.8 [4.3 15.8]	8.5 [4.7 15.1]	6.9 [4.2 13.9]	7.4 [4.1 15.4]	0.271
**Urinary ACR ≥ 30 mg/g, %**	11.1	11.1	10.8	8.5	14.0	0.115
**CKD, %**	15.9	14.9	15.2	12.9	20.5	0.044

Values for categorical variables are given as percent; for continuous variables, as mean ± standard deviation; for CRP and urinary ACR, as median [interquartile range].

eNEAP, estimated net endogenous acid production; CVD, cardiovascular disease; HEPA, health-enhancing physical activity; BMI, body mass index, BP, blood pressure, CRP, C-reactive protein, eGFR, estimated glomerular filtration rate; ACR, albumin creatinine ratio; CKD, chronic kidney disease.

Dietary patterns according to eNEAP quartile are shown in Table 2. Compared with those in the lowest eNEAP quartile (Q1, reference group), individuals in the higher eNEAP quartiles (Q2, Q3, Q4) had lower potassium intake and higher protein intake (*P* for trend < 0.001). In the higher eNEAP quartiles (Q2, Q3, Q4), protein intake was increased, but potassium intake was greatly decreased compared to the lowest eNEAP quartile (Q1). eNEAP was correlated with potassium intake (r = -0.410, *P* < 0.001), but not correlated with protein intake (r = -0.004, *P* = 0.879) (S1 Fig).

**Table 2:** Dietary characteristics by dietary eNEAP quartiles.

Dietary parameter	All	eNEAP, mEq/day				*P* for trend
		Q1 (1.1 to 33.3)	Q2 (33.4 to 45.5)	Q3 (45.6 to 60.5)	Q4 (60.6 to 219.1)	
**Number of patients**	1,369	343	342	342	342	
**eNEAP, mEq**	50.4±26.5	25.5±7.0	39.9±3.5	52.2±4.0	84.1±29.3	<0.001
**Protein intake, g/day**	44.4±25.3	35.3±19.3	48.6±23.4	49.7±22.5	44.0±31.8	<0.001
**Protein intake, g/Kg/day**	0.73±0.45	0.60±0.34	0.80±0.41	0.81±0.37	0.72±0.60	0.001
**Potassium intake, mg/day**	1733±990	2067±1048	2059±973	1700±776	1105±814	<0.001
**Protein:Potassium ratio**	1112±486	656±128	919±65	1145±74	1731±538	<0.001
**Sodium intake, mg/day**	1956±1420	2353±1608	2395±1442	1910±1101	1165±1107	<0.001
**Calorie intake, Cal/day**	1283±678	954±619	1378±642	1464±632	1335±701	<0.001

eNEAP, estimated net endogenous acid production.

### eNEAP and CKD

The participants in the highest eNEAP quartile (Q4) had higher odds of CKD compared to those in the lowest eNEAP quartile (Q1); the OR (95% CI) was 1.47 (1.00–2.19) in the crude model. In the multivariate adjustment in model 2, the participants in higher eNEAP quartiles (Q2, Q3, Q4) exhibited higher odds of CKD compared to those in the lowest eNEAP quartile (Q1); ORs (95% CI) were 1.47 (0.78–2.76), 1.66 (0.85–3.23), and 2.30 (1.16–4.60) respectively (*P* for trend = 0.019) (Table 3). There was a stronger association between high dietary eNEAP and CKD after full adjustment.

**Table 3:** Association of CKD with dietary eNEAP, protein intake, and potassium intake quartiles.

	Odds ratio (95% confidence interval)				*P* for trend
	Quartile 1	Quartile 2	Quartile 3	Quartile 4	
**eNEAP quartile increase**					
**Crude**	1.00 (reference)	1.02 (0.68–1.56)	0.85 (0.55–1.30)	1.47 (1.00–2.19)	0.102
**Model 1**	1.00 (reference)	1.07 (0.69–1.66)	0.88 (0.55–1.42)	1.45 (0.90–2.34)	0.189
**Model 2**	1.00 (reference)	1.47 (0.78–2.76)	1.66 (0.85–3.23)	2.30 (1.16–4.60)	0.019
**Protein intake quartile increase**					
**Crude**	1.00 (reference)	0.64 (0.42–0.96)	0.85 (0.58–1.26)	0.64 (0.42–0.97)	0.104
**Model 1**	1.00 (reference)	0.61 (0.38–1.01)	0.77 (0.45–1.34)	0.55 (0.27–1.16)	0.260
**Model 2**	1.00 (reference)	0.53 (0.27–1.02)	0.68 (0.32–1.46)	0.56 (0.21–1.47)	0.444
**Potassium intake quartile increase**					
**Crude**	1.00 (reference)	0.69 (0.46–1.03)	0.73 (0.49–1.08)	0.64 (0.43–0.97)	0.046
**Model 1**	1.00 (reference)	0.68 (0.44–1.05)	0.71 (0.44–1.17)	0.65 (0.34–1.22)	0.196
**Model 2**	1.00 (reference)	0.52 (0.28–0.95)	0.50 (0.26–0.96)	0.50 (0.21–0.99)	0.050

Model 1: Adjusted for age, sex, total caloric intake, dietary sodium intake; Model 2: model 1+ body mass index, smoking, education state, health-enhancing physical activity; hypertension, diabetes, hyperlipidemia, cardiovascular disease.

CKD, chronic kidney disease; eNEAP, estimated net endogenous acid production.

**Fig 1:**
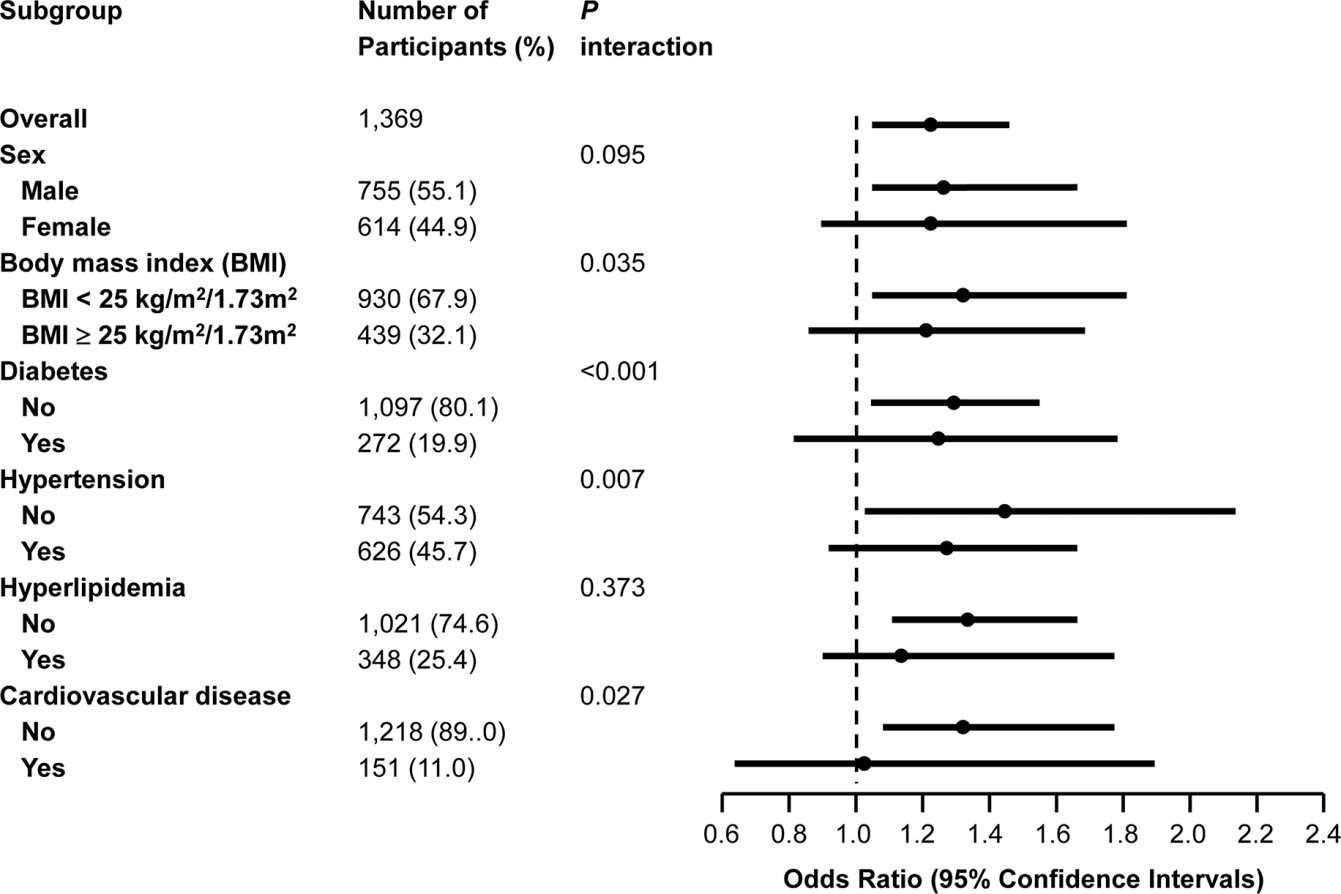
Forest plot of dietary estimated net endogenous acid production (eNEAP) and chronic kidney disease in overall participants and subgroups. Odds ratios (95% confidence intervals) were per quartile of eNEAP. Odds ratios were adjusted for age, sex, total caloric intake, dietary sodium intake, body mass index, smoking, education state, health-enhancing physical activity, hypertension, diabetes, hyperlipidemia, and cardiovascular disease (Model 2).

In a multivariate adjusted stratified analysis, the relationship between eNEAP and CKD was affected by the presence of obesity, diabetes, hypertension, and CVD. The association appeared to be stronger in those without obesity, diabetes, hypertension, and CVD; ORs (95% CI) were 1.37 (1.04–1.81), 1.33 (1.01–1.73), 1.48 (1.01–2.20), and 1.35 (1.06–1.71) respectively (*P* for interaction < 0.05). The tests for interaction were not statistically significant according to the sex and hyperlipidemia (Fig 1).

### Protein intake, potassium intake and CKD

In the analysis of nutrients to relate to eNEAP, similar patterns were observed for the association between potassium intake and CKD. The participants in the highest potassium intake quartile (Q4) had lower odds of CKD compared with those in the lowest potassium intake quartile (Q1); OR (95% CI) was 0.64 (0.43–0.97) in crude model. In the multivariate adjustment in model 2, the participants in the higher potassium intake quartiles (Q2, Q3, Q4) had lower odds of CKD compared to those in the lowest potassium quartile (Q1); ORs (95% CI) were 0.52 (0.28–0.95), 0.50 (0.26–0.96), and 0.50 (0.21–0.99) respectively (*P* for trend = 0.050) (Table 3). Protein intake was not associated with CKD.

## Discussion

In this cross-sectional study of elderly Koreans, a dietary pattern of high eNEAP was associated with CKD. In the two nutrient components of the eNEAP prediction equation, high potassium intake was negatively associated with CKD, but protein intake was not associated with CKD. This association was stronger after multiple adjustment models for sociodemographic factors, dietary factors, and comorbidities. In the stratified analysis, the associations between eNEAP and CKD were more consistent in the elderly adults without diabetes, hypertension, and CVD.

Contemporary Western-style diets are acid producing. Dietary acid producing is determined by the balance between acid production by sulfur-containing amino acid from protein and bicarbonate generation from alkali-containing potassium salts. Recent studies have showed that the Western-style diet would be potentially a risk factor for impaired kidney function and CKD [5]. Dietary patterns vary according to race, region, and age group. The characteristics of the Korean diet are high consumption of vegetables, moderate to high consumption of legumes and fish, and low consumption of red meat [19]. In the Korea National Health and Nutrition Examination Survey (KNHANES) 1998–2010, approximately 40% of adults still followed a Korean dietary pattern and the popularity of the Western dietary pattern was approximately 20% [19]. In the KNHANES 2011–2012, the prevalence of CKD was estimated as 7.9% in adults (over 19 years old), but was more common in the older population (26.8% over 68 years old) [20]. In this study, we therefore evaluated the association between dietary acid load and CKD in community-dwelling elderly Koreans. We also examined their relation to protein intake and potassium intake.

Previous studies on the association between dietary acid load and health benefits are inconclusive. In a meta-analysis, the association between dietary acid loads and osteoporotic bone disease was not supported [21]. While a prospective cohort study reported that dietary acid load was associated with the development of type 2 diabetes [22], the impact of high acid dietary load on insulin resistance and diabetes remain unclear in recent review [23]. However, recent studies in renal disease suggest that dietary acid load is associated with CKD and that base administration slows the progression of poor outcomes in CKD patients with metabolic acidosis [7]. The Atherosclerosis Risk in Communities (ARIC) study was a community-based observational study of middle aged adults in the US demonstrating that dietary acid load was associated with incident CKD [8]. In the cross-sectional study of the National Health and Nutrition Examination Survey (NHANES) 1999–2004 in US adults, a higher eNEAP was associated with albuminuria and lower eGFR. In an observational study of adults with CKD, a high dietary acid load was associated with increased risk of ESRD [24]. In this study of 1,369 community-dwelling elderly Korean people, high eNEAP was independently associated with CKD.

Although the MDRD study, the largest randomized controlled trial to examine protein restriction in CKD, showed inconclusive results [9], low protein diet has been recommended to delay initiation of dialysis or control metabolic derangement in CKD. In a prospective study using Singapore Chinese Health Study, red meat intake may increase the risk of ESRD, but soy and legumes intake may reduce the incidence of ESRD [25]. This suggested that protein from plant-based compared with red meat sources may have different effects on the renal function. In present study of elderly Koreans, protein intake was not associated with CKD. The possible reason is that the elderly Koreans intake relatively low protein diet and sources of protein intake more from plant protein than from red meat in Korean diet.

Potassium was identified as a shortfall nutrient by the Dietary Guidelines for Americans Committee [26] and potassium intake reduced blood pressure and influenced the risk of cardiovascular disease [11,12]. A cross-sectional study of the NHANES reported higher potassium intake was associated with lower odds of CKD among US adults [27]. The Prevention of Renal and Vascular End-Stage Disease (PREVEND) study showed that low urinary potassium excretion was associated with increased risk of developing CKD [28]. Several studies indicated that low urinary potassium excretion was associated with CKD progression and suggested that low potassium intake could increase the risk of CKD progression [13,27–29]. In the present study, potassium intake was negatively associated with CKD. This suggested that potassium is a major factor of NEAP and may have a protective role in CKD development in elderly Koreans. This should be corroborated through the longitudinal studies or clinical trials to examine the effects of dietary potassium intake on CKD.

There is no substantial evidence that low dietary acid load and high potassium intake protect kidney health. The proposed mechanisms that dietary acid load induces the renal damage include tubular toxicity of high intramedullary ammonium [30] and activation of the renin-angiotensin system by high dietary acid [6,31]. Potassium intakes are associated with endothelium–dependent vasodilatation via the sodium-potassium pump [11]. Dietary fiber content in high potassium diet such as fruit and vegetables may reduce serum creatinine due to increase creatinine degradation by intestinal bacteria and improves variety of CKD risk factors including dampening glycemic excursions, improving lipid metabolism, and improving blood pressure [32].

Our study has several limitations. First, the cross-sectional design limits the possibility of causal inferences as we cannot rule out reverse causation or residual confounding. Second, assessment of dietary intake by self-report FFQ is prone to reporting bias and measurement error. We couldn’t analyze the data separated from animal protein and plant protein. Third, the present study population primarily consisted of relatively healthy elderly Koreans in good economic state, and thus our findings may not be generalizable to other populations. Fourth, a one-time measurement of eGFR and albuminuria could lead to a misclassification bias of CKD. However, we examined the association after selecting relatively healthy elderly adults and controlling for many potential confounders of CKD and dietary factors. KSCS collected data with extensive quality control and standardization. The 103-item FFQ of Korean has been clinically useful in several studies of KSCS cohorts [33,34].

In conclusion, we demonstrated an association between dietary acid load and CKD in elderly adults. The associations were stronger in subgroups without obesity, diabetes, hypertension, and CVD. Among the nutrients related to dietary acid load, potassium intake was associated with CKD. Further studies are needed to assess the consistency of these results in prospective cohort studies or clinical trials ultimately to provide dietary prevention of CKD.

## Supporting information

S1 Fig Correlations between estimated net endogenous acid production (eNEAP) and nutrient intakes. (A) protein intake, and (B) potassium intake. The straight-line represents the best-fit lines obtained by linear regression analysis.
(TIF)
