# Supplementary figures and images for: Dietary acid load and chronic kidney disease in elderly adults: Protein and potassium intake
Source: PLoS One. 2017 Sep 27;12(9):e0185069. doi: 10.1371/journal.pone.0185069 (PMC5617182; doi:10.1371/journal.pone.0185069)

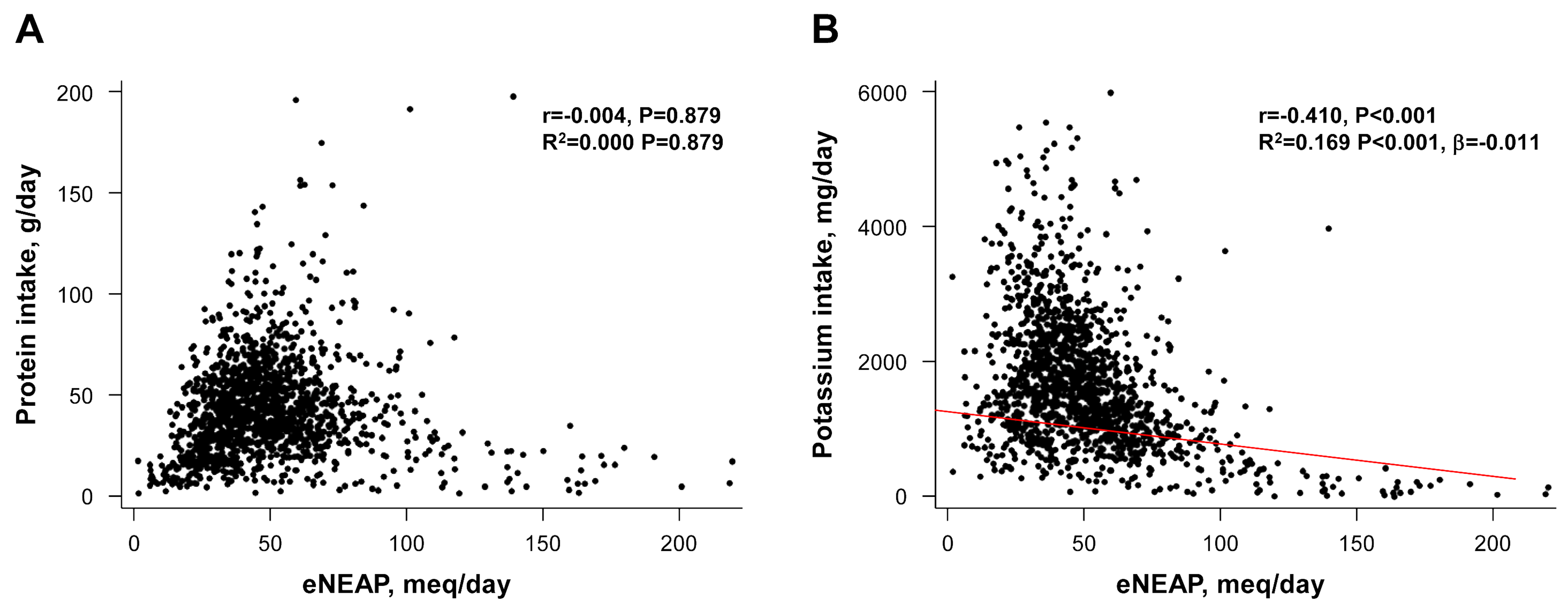

Supplement: S1 Fig — (A) protein intake, and (B) potassium intake. The straight-line represents the best-fit lines obtained by linear regression analysis. (TIF) [file pone.0185069.s001.tif]
